# Viral Metagenomic Analysis Displays the Co-Infection Situation in Healthy and PMWS Affected Pigs

**DOI:** 10.1371/journal.pone.0166863

**Published:** 2016-12-01

**Authors:** Anne-Lie Blomström, Caroline Fossum, Per Wallgren, Mikael Berg

**Affiliations:** 1 Department of Biomedical Sciences and Veterinary Public Health, Section of Virology, Swedish University of Agricultural Sciences, Uppsala, Sweden; 2 Department of Biomedical Sciences and Veterinary Public Health, Section of Immunology, Swedish University of Agricultural Sciences, Uppsala, Sweden; 3 National Veterinary Institute (SVA), Uppsala, Sweden; Universitatsmedizin Greifswald, GERMANY

## Abstract

The development of high-throughput sequencing technologies have allowed the possibility to investigate and characterise the entire microbiome of individuals, providing better insight to the complex interaction between different microorganisms. This will help to understand how the microbiome influence the susceptibility of secondary agents and development of disease. We have applied viral metagenomics to investigate the virome of lymph nodes from Swedish pigs suffering from the multifactorial disease postweaning multisystemic wasting syndrome (PMWS) as well as from healthy pigs. The aim is to increase knowledge of potential viruses, apart from porcine circovirus type 2 (PCV2), involved in PMWS development as well as to increase knowledge on the virome of healthy individuals. In healthy individuals, a diverse viral flora was seen with several different viruses present simultaneously. The majority of the identified viruses were small linear and circular DNA viruses, such as different circoviruses, anelloviruses and bocaviruses. In the pigs suffering from PMWS, PCV2 sequences were, as expected, detected to a high extent but other viruses were also identified in the background of PCV2. Apart from DNA viruses also RNA viruses were identified, among them were a porcine pestivirus showing high similarity to a recently (in 2015) discovered atypical porcine pestivirus in the US. Majority of the viruses identified in the background of PCV2 in PMWS pigs could also be identified in the healthy pigs. PCV2 sequences were also identified in the healthy pigs but to a much lower extent than in PMWS affected pigs. Although the method used here is not quantitative the very clear difference in amount of PCV2 sequences in PMWS affected pigs and healthy pigs most likely reflect the very strong replication of PCV2 known to be a hallmark of PMWS. Taken together, these findings illustrate that pigs appear to have a considerable viral flora consisting to a large extent of small single-stranded and circular DNA viruses. Future research on these types of viruses will help to better understand the role that these ubiquitous viruses may have on health and disease of pigs. We also demonstrate for the first time, in Europe, the presence of a novel porcine pestivirus.

## Introduction

Viral metagenomics and high-throughput sequencing technology have allowed the detection and characterisation of novel viruses. The technology provides the possibility to investigate the aetiology of diseases of unknown cause as well as to characterise the virome of healthy individuals or of, for example, the environment [1, 2]. It has for instance showed that different wildlife reservoirs such as bats have a very diverse virome including mammalian viruses of both human and animal relevance [3]. Metagenomic studies have also demonstrated a high diversity of viruses in mammalians such as the pig [4, 5]. A study utilizing high-throughput sequencing to characterise the fecal virome of diarrheic and healthy piglets showed that healthy piglets shed an average of 4.2 different mammalian viruses and an average of 5.4 mammalian viruses were shed by the diarrheic piglets [5]. Thus, revealing a high frequency of co-infections in these pigs. Similar results have been seen in humans as well as in other animal species, including horses [6] and turkeys [7], indicating that the presence of several viruses in an individual is common.

Postweaning multisystemic wasting syndrome (PMWS) is a multifactorial disease associated with high replication levels of porcine circovirus type 2 (PCV2), primarily in the lymph nodes, leading to increased size of the lymph nodes and acute wasting with a rapid loss of weight. Occasionally also jaundice, paleness and respiratory problems, are recorded [8]. However, PCV2 is an ubiquitous virus in pig populations around the world. It has been associated with a number of porcine circovirus related diseases apart from PMWS, but it is also present in healthy herds [9]. In affected herds the rate of PCV2 infection is high but despite this only 5–30% of infected pigs develop clinical signs of PMWS [10]. The triggers to the onset of disease remains unclear but experimental settings in snatch farrowed colostrum deprived piglets have demonstrated that co-infections with PCV2 and for instance parvovirus or porcine reproductive and respiratory syndrome virus induce PMWS in contrast to infection with PCV2 alone [11–13].

We have in a previous study used metagenomic to investigate lymph node samples from pigs suffering from PMWS in order to identify additional viral agents in these samples. A porcine bocavirus was found in the background of PCV2, as were Torque Teno sus virus 1 and 2 (TTSuV1 and 2) [14], but if these viruses are involved in the development of PMWS is at present unknown. It is notable that all these viruses also were demonstrated in healthy pigs. However, that study only focused on DNA viruses and the number of sequence reads was limited (approximately 9000 reads). Therefore, we have performed an extended investigation where viral metagenomic was used to characterise the entire virome (RNA and DNA viruses) of lymph node samples from individuals suffering from PMWS as well as from healthy pigs.

## Material and Methods

### Samples

Previously collected lymph nodes, stored at -80°C, were used in this study; samples 1 and 2 were subiliac lymph nodes collected from two healthy pigs bought from an SPF herd [15] in February 1993. These pigs had been injected intradermally with killed and gluteraldehyde-fixed Aujeszky’s Disease virus (ADV) 24 h before been euthanasia. It is also notable that the herd of origin was seronegative to PCV2 in February 1993 but not in May 1993 [16, 17] and that PMWS has never been diagnosed in this herd. Sample 3 and 4 were mesenteric lymph nodes collected from healthy animals from the same SPF herd but nine years later in 2004. PCV2a, bocavirus and TTSuV1 had previously been demonstrated in these pigs, and in pig 4 also TTSuV2 had been demonstrated [18, 19]. Samples 5 and 6 were mesenteric lymph nodes collected from PMWS affected pigs in a conventional pure bred Landrace herd in 2004, and PCV2b had previously been demonstrated in these pigs [19]. Sample 7 and 8 were mesenteric lymph nodes collected in 2007 from conventional fattening pigs (Hampshire x Landrace-Yorkshire) suffering from acute PMWS. PCV2b had previously been demonstrated in these pigs [20]. The study was approved by the Ethical Committee in Uppsala, Sweden (certificate C39/14)

### Sample preparation

The tissues were mechanically homogenized in 1.5 ml of 1x DNase buffer (Roche) and centrifuged at 4000 rpm for 10 minutes. The supernatant was aliquoted to 200 μl volumes and 100U DNase (Roche) and 2 μg of RNase A (Invitrogen) was added to each aliquot. After a short vortex the samples were incubated for 2 hours at 37°C prior to DNA and RNA extraction.

### RNA and DNA extraction

One half of the sample was used for DNA extraction and the other half for RNA extraction. DNA was extracted using QIAamp DNA mini kit (Qiagen) according to the manufacturers instruction and the DNA was eluted in 50 μl AE. RNA was extracted using a combination of Qiazol (Qiagen) and RNeasy mini kit and eluted in 30 μl RNase-free H_2_0.

### Nucleic acid labelling, random PCR and high throughput sequencing

The RNA was labelled with the PCR primer tag-sequence through the cDNA synthesis while the DNA was labelled through a Klenow Fragment (3′ → 5′exo^-^) reaction. The cDNA and DNA was amplified using AmpliTaq Gold polymerase as described elsewhere [21]. Prior to sequencing, the RNA and DNA amplified product from each respective individual were pooled. The library preparation, emulsion PCR and sequencing was performed by the SNP&SEQ Technology Platform in Uppsala. The samples were sequenced using the Genome Sequencer FLX, Roche/454, all samples were sequenced together on one Picotiter plate according to standard procedures. The raw sequence data have been uploaded to the Sequence Read Archive (NCBI)–accession number SRP076383.

### Data analysis

The quality of the sequences from the 454-sequening run was checked using CLC-genomic workbench. The program was also used for *de novo* assembly and both the created contigs as well as the remaining singletons were then subjected to blastn (database nt). The sequences that did not render a blastn result were subjected to blastx (database nr) searches. The Camera portal was used for all the blast searches [22]. An e-value of 10^−4^ was used as a cut-off value to enable detection of divergent viruses. For the taxonomic classification the top blast hit was used.

### Confirmation and genetic analysis

Primers (Table 1) were designed against a number of the viral hits from the blastn and blastx searches using the respective contigs and singletons as template. PCRs were run according to the following protocol: 1X PCR buffer, 2.5 mM MgCl2, 0.2 mM of each nucleotide, 0.4 mM forward primer, 0.4 mM reverse primer and 1.25U AmpliTaq Gold DNA polymerase. Amplification was initiated with a 12 minute heating step at 95°C followed by 40 cycles of 30 s at 95°C, 30 s at 58°C and 90 s at 72°C. The amplification was finalised with an elongation step at 72°C for 10 minutes and the PCR products were run on an agarose gel. The positive PCR products were sent for Sanger sequencing (Macrogen). SeqMan (Lasergene 9.1, DNASTAR) was used to edit and assemble the sequences. Bioedit was used to compare the sequences to previously published related viruses and MEGA6 [23] was used to construct neighbour-joining trees with a bootstrap value of 1000. The sequences generated were deposited in GenBank: accession numbers KU727772-4.

**Table 1:** Primers used for verification of the sequence annotation (5’-3’).

	Forward primer	Reverse primer
K2/SWE/circoviridae	GCCTCGTGTTTTGATGCCGCAGGA	GAGGCCGCTTCATCATCCACTGC
APPV1	GCAAAGATGCCCCTGATTGT	CTCCAGTTTGTCTAACTCGTCT
PBoV3	CGTCACGGTCGACCAAGTC	AGTCAGACTGCAAAGAGTCGA

## Results

### 454 sequencing results

On average, around 100 000–230 000 sequences were obtained for each sample with an average of 260 bp in length. However, only 4559 sequences were obtained from pig 5.

### Sequence annotation

**Fig 1:**
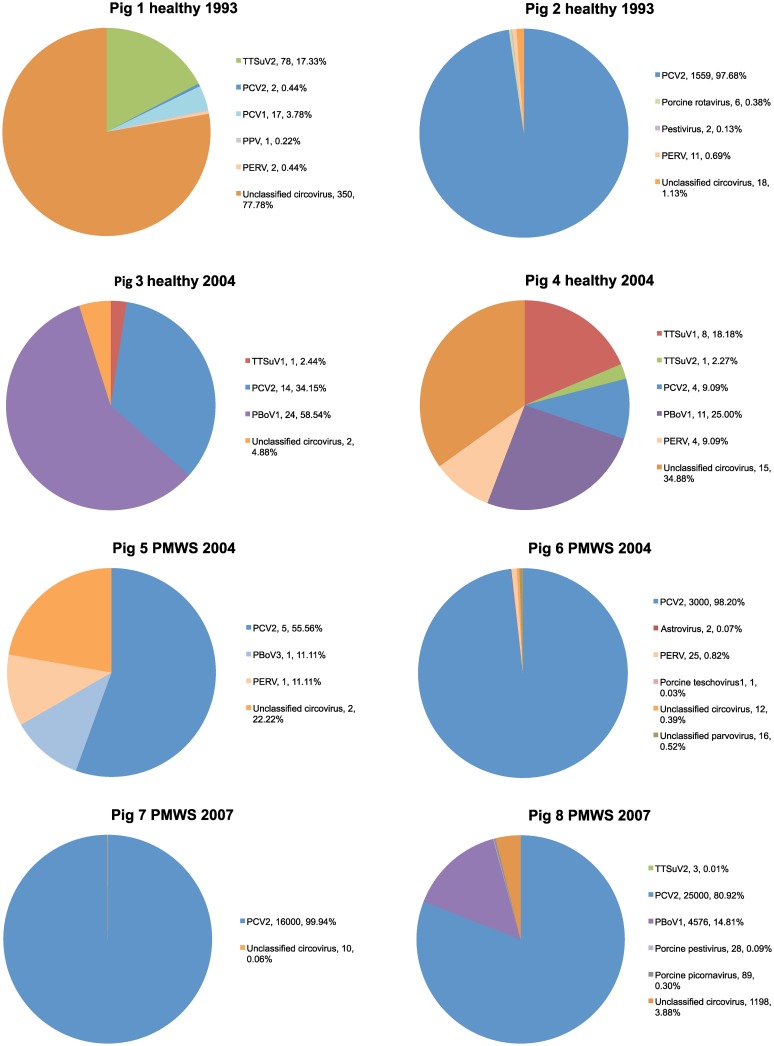
The virome of each lymph node. The legend shows the total number of reads (including those making up a contig) that belonged to each respective virus as well as the percentage of each virus in relation to all viral sequences identified in the specific sample.

The sequences were annotated through blastn and blastx searches using the Camera portal [22]. In all the datasets 70–95% of the sequences were of Eukaryotic origin indicating that the nuclease treatment had not worked well and/or that there was a low amount of viruses in these samples. However, despite this, viral sequences were identified in all of the pigs. The virome of each lymph node sample is summarized in Fig 1. A large number of sequences showed closest similarity to different single-stranded DNA viruses but also a number of RNA viruses were identified. It should be noted that not all sequences could be annotated using blast. The origin of these sequences is thus unknown and further investigations are needed to know if any of these could be viruses too divergent to be identified through blast analysis.

In samples 1 and 2, collected 10 years prior to the first outbreak of PMWS in Sweden, a majority of the identified viral sequences belonged to different single-stranded DNA viruses. In pig 1, both PCV1 and PCV2 were identified as well as other unclassified circoviruses. The majority of the unclassified circovirus hits, showed closest similarity to circovirus NW2 identified in pork product in the USA [24, 25]. The identities to the available GenBank sequences were on nucleotide level 92–100% covering both the replicase and the capsid protein genes. From pig 2, a vast majority of the viral sequences matched to PCV2. Apart from different circovirus, also sequences showing similarities to, for example, parvovirus and pestivirus were identified in pig 1 and 2.

In lymph nodes 3 and 4, collected from healthy SPF pigs in 2004 a vast diversity of viruses was identified (Fig 1). In both these pigs, porcine bocavirus (PBoV), PCV2, anellovirus (TTSuV1 and TTSuV2 (only pig 4)) and unclassified circoviruses were identified. The PBoV in these pigs belonged to the same genogroup as that previously described in Sweden, as were the TTSuVs [14, 18].

A different picture was seen in the lymph nodes from the pigs affected by PMWS, i.e. in pig 5 and 6 from 2004 and in pig 7 and 8 from 2007 (Fig 1). In these samples PCV2 sequences was identified to a much higher degree and was the absolute dominating virus detected. However, many of the viruses identified in the samples from the healthy pigs were also identified in the background of PCV2 of these PMWS-affected pigs such as, for example, different unclassified circular DNA viruses but also parvovirus, astrovirus, teschovirus, parvovirus and pestivirus was identified. Very few sequences were obtained from pig 5, and therefore those viruses observed in the other samples may have escaped detection. However, also in this sample bocavirus, circovirus and porcine endogenous retroviral sequences were identified.

### Verification and sequence analysis of discovered viruses

We selected three of the identified viruses for further analysis. Main focus was on those that had not previously been detected in Sweden and/or that showed a divergence to previously described viruses.

**Fig 2:**
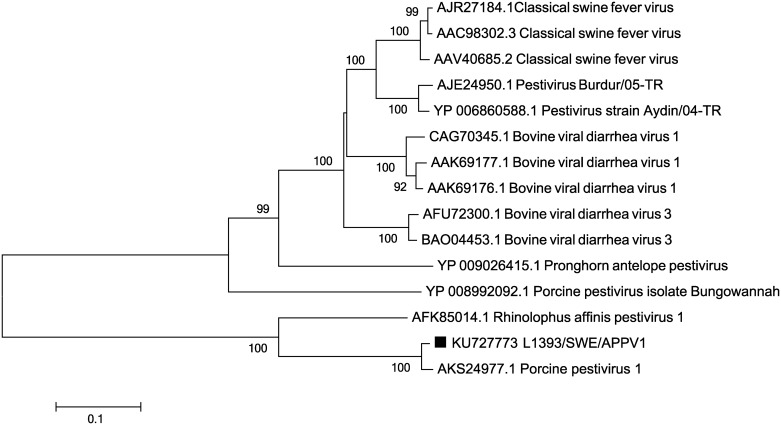
Phylogenetic relationship between the identified pestivirus and available pestiviruses in GenBank using parts of the polyprotein (311 amino acids). The evolutionary history was inferred using the Neighbour-joining method, with a bootstrap test of 1000 replicates. The evolutionary distances were computed using the p-distance method. The viral sequence characterised in the present study (KU727773) is marked with ■.

Pestiviral sequences was present in two of the samples, in particular in the sample from pig 8. Using PCR a region of around 1500 bp was amplified from this virus. This sequence showed closest similarity to an atypical porcine pestivirus (APPV) (also called Porcine pestivirus 1) that was detected in pigs in the USA in 2015 [26]. On nucleotide level 90% identity was observed and on amino acid level 98%. The second most similar virus sequence available was a pestivirus identified in the bat species *Rhinolophus affinis*—Rhinolophus affinis pestivirus 1 (Ra-PestV-1) [3] and to this a 70% amino acid sequence similarity was observed. To other porcine pestiviruses, such as the isolate Bungowannah, the protein identity was only 36%. Phylogenetic analysis (Fig 2) confirmed that the virus identified in this particular study, APPV (i.e. Porcine pestivirus 1) and Ra-PestV-1 group away from other known pestiviruses.

**Fig 3:**
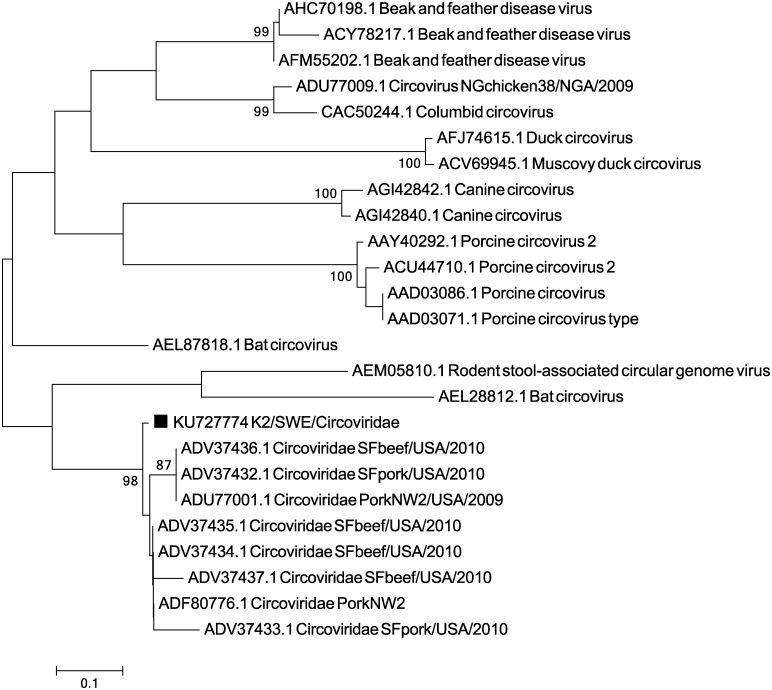
Phylogenetic relationship between the circovirus with closest similarity to circovirus Pork NW2 and related circoviruses using parts of the rep protein (141 amino acids). The evolutionary history was inferred using the Neighbour-joining method, with a bootstrap test of 1000 replicates. The evolutionary distances were computed using the p-distance method. The viral sequence characterised in the present study (KU727774) is marked with ■.

Apart from PCV2/1 and TTSuV1/2 unclassified single-stranded circoviruses were identified through the annotation. The most prevalent of these were viruses with similarity to a circovirus originally identified in pork products in the US in 2009 (PorkNW2) [25]. PCR and Sanger sequencing confirmed the presence of this virus. The virus phylogenetically clustered (Fig 3) with the viruses that Li *et al*. (2010) identified in pork and beef and the protein sequence identity was high to those isolates (97–99%).

**Fig 4:**
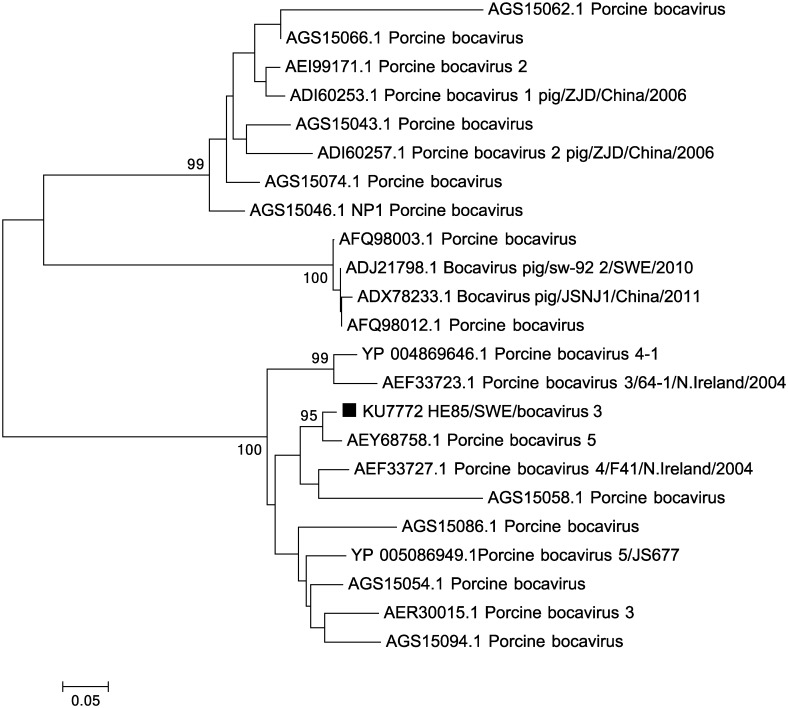
Phylogenetic relationship between the identified PBoV 3 and related porcine bocaviruses using parts of the NP protein (162 amino acids). The evolutionary history was inferred using the Neighbour-joining method, with a bootstrap test of 1000 replicates. The evolutionary distances were computed using the p-distance method. The viral sequence characterised in the present study (KU727772) is marked with ■.

The 454-sequence analysis indicated that, apart from the porcine bocavirus (PBoV group 1) previously demonstrated in Swedish pig [14, 18], a second bocavirus species/group (PBoV group 3) were present in the sample collected from pig 5. This was verified by a specific PCR targeting this virus. The sequence analysis showed a 93% nucleotide sequence identity, over the amplified 685bp region covering parts of the NP and VP1 gene, to an isolate from Northern Ireland [27]. Phylogenetic analysis grouped the bocavirus from this study with PBoVs members of group 3 rather than with the bocavirus that have previously been described in Sweden (Fig 4).

## Discussion

In this study, we show that a number of different single-stranded DNA viruses are circulating in pig populations in Sweden apart from the previously well-known PCV2, TTSuV1 and 2. In many cases a high level of viral co-infection appeared to be present in different individuals, this was especially apparent in the healthy individuals. Apart from the previously mentioned PCV2, TTSuV1 and 2, we discovered a virus showing high sequence identity (almost 100%) to a circovirus identified in pork products in the USA (Li 2010, 2011). This virus was identified in two lymph nodes from healthy pigs and from one PMWS pig. As the samples were collected in 1993, 2004 and 2007 this virus has apparently been present in Swedish pig herds for at least two decades.

Linear single-stranded DNA viruses, including PBoV1 that have previously been identified in Swedish pig herds [14, 18], were detected in the different samples. PBoV1 was identified in two of the healthy pigs as well as in one of the PMWS pigs (pig 8). Despite the high number of PCV2 sequence reads (>25000) as much as 4576 reads belonged to PBoV1 in this pig. One sample (pig 5) had one sequence read annotating to a porcine bocavirus belonging to genogroup 3. This was verified by PCR showing that several bocavirus genogroups are present in Swedish pig herds. PBoV are widespread and at least five different PBoV species have been detected in Europe, Asia, America and Africa [5, 14, 18, 27–29]. A study from USA showed that a number of genetically diverse PBoVs co-circulate and that there is a high frequency of co-infections of several PBoV species within individuals [30]. In order to achieve a similar complete picture of the PBoV status in Sweden, further studies for all the known PBoV species are needed. There was also a more divergent parvovirus found among the sequences in one of the samples (pig 1) showing 61% sequence identity to the VP1 gene of porcine parvovirus indicating that there might be an additional parvovirus present in this sample.

Not only DNA viruses were identified but also different RNA viruses. Pig 6 had sequences that showed 91% amino acid and 87% nucleotide sequence identity to a part of the VP-gene of porcine teschovirus 1 (PTV-1). This is a member of the *Picornaviridae* family. PTV-1 infections are often asymptomatic but can occasionally lead to clinical disease in the form of encephalomyelitis in pigs [31] However, no such clinical signs were present in pig 6 indicating an asymptomatic infection. Another RNA virus identified in one of the samples (pig 8) showed closest similarity to the highly divergent APPV that was discovered in pigs in the USA in 2015 and that appear to be widespread in USA but that has to date not been reported in other countries [26]. These two viruses only show low identity to previously described porcine pestivirus and at the time of the discovery of APPV it showed closest similarity to Ra-PestV-1, a pestivirus belonging to the *Flaviviridae* family identified in a bat metagenomic study from China [3].

There was an apparent difference in the amount of PCV2 sequences detected in the PMWS pigs compared to the healthy individuals. While high numbers of PCV2 sequences were found in PMWS samples, only a few were found in the lymph nodes from healthy individuals (with the exception of pig 2), although this is not a quantitative method this difference most likely reflect the massive PCV2 replication always seen in PMWS pigs. In fact, the percentage of PCV2 sequences was not higher than other viral sequences in most of the samples collected from healthy pigs. Many of the viruses found in healthy individuals were also present in the PMWS samples.

## Conclusions

Taken together, this study show for the first time the presence of the atypical porcine pestivirus APPV outside of the US and PBoV 3 in Sweden. Our study also shows that there is a high co-infection situation in healthy pigs, as well as in pigs with PMWS. This is in concurrence with other studies that have shown simultaneous identification of different RNA and DNA viruses in porcine faeces samples [4, 5]. Future studies are needed not only to genetically characterise these viruses and perform screening studies for the different viruses in a larger sample sets but also to study the role of these virus alone and during co-infection situations with the aims of elucidating how/if these viruses interact with the host immune system to validate their role in the pathogenesis of diseases and secondary infections.
